# Barlow Twins deep neural network for advanced 1D drug–target interaction prediction

**DOI:** 10.1186/s13321-025-00952-2

**Published:** 2025-02-05

**Authors:** Maximilian G. Schuh, Davide Boldini, Annkathrin I. Bohne, Stephan A. Sieber

**Affiliations:** 1 Chair of Organic Chemistry II, Department of Bioscience, TUM School of Natural Sciences, Center for Functional Protein Assemblies (CPA), Technical University of Munich, Ernst-Otto-Fischer Str. 8, 85748 Garching bei München, Bavaria Germany; 2 Chair of Biochemistry, Department of Bioscience, TUM School of Natural Sciences, Center for Functional Protein Assemblies (CPA), Technical University of Munich, Ernst-Otto-Fischer Str. 8, 85748 Garching bei München, Bavaria Germany

**Keywords:** Machine learning, Deep neural network, Drug discovery, Drug–target interactions

## Abstract

**Abstract:**

Accurate prediction of drug–target interactions is critical for advancing drug discovery. By reducing time and cost, machine learning and deep learning can accelerate this laborious discovery process. In a novel approach, BarlowDTI, we utilise the powerful Barlow Twins architecture for feature-extraction while considering the structure of the target protein. Our method achieves state-of-the-art predictive performance against multiple established benchmarks using only one-dimensional input. The use of our hybrid approach of deep learning and gradient boosting machine as the underlying predictor ensures fast and efficient predictions without the need for substantial computational resources. We also propose the use of an influence method to investigate how the model reaches its decision based on individual training samples. By comparing co-crystal structures, we find that BarlowDTI effectively exploits catalytically active and stabilising residues, highlighting the model’s ability to generalise from one-dimensional input data. In addition, we further benchmark new baselines against existing methods. Together, these innovations improve the efficiency and effectiveness of drug–target interactions predictions, providing robust tools for accelerating drug development and deepening the understanding of molecular interactions. Therefore, we provide an easy-to-use web interface that can be freely accessed at https://www.bio.nat.tum.de/oc2/barlowdti.

**Scientific contribution:**

Our computationally efficient and effective hybrid approach, combining the deep learning model Barlow Twins and gradient boosting machines, outperforms state-of-the-art methods across multiple splits and benchmarks using only one-dimensional input. Furthermore, we advance the field by proposing an influence method that elucidates model decision-making, thereby providing deeper insights into molecular interactions and improving the interpretability of drug-target interactions predictions.

**Graphical Abstract:**

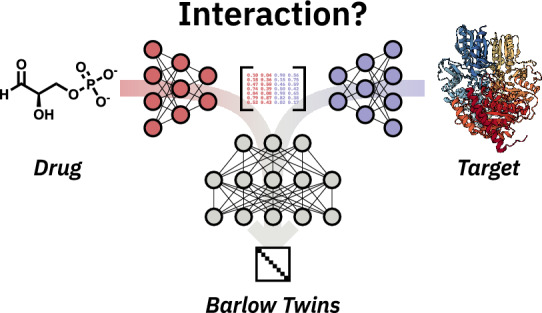

**Supplementary Information:**

The online version contains supplementary material available at 10.1186/s13321-025-00952-2.

## Introduction

Studying drug-target interactions (DTI) is crucial for understanding the biochemical mechanisms that govern how molecules interact with proteins [1]. Key challenges in drug discovery are the identification of proteins that can be used as targets for the treatment of diseases [2]. To achieve the desired therapeutic effects, the discovery of molecules that interact with and activate or inhibit target proteins is essential [3–5].

Recent advances in computational methods have transformed the drug discovery landscape, providing robust tools for cost-effective exploration of the chemical space. These *in silico* approaches facilitate the prediction and analysis of drug-target interactions, aiding in the identification of potential drug candidates and their corresponding protein targets [6–11]. The use of computational techniques allows researchers to gain a comprehensive understanding of the molecular mechanisms underlying drug-target interactions, thereby accelerating the drug discovery process and minimising reliance on traditional, resource-intensive experimental methods [12, 13]. Different methods have been used to understand how drugs interact with target proteins. These methods are grouped into three main categories: structure-agnostic, structure-based and complex-based.

Structure-agnostic approaches use one-dimensional (1D) representations like molecule simplified molecular-input line-entry system (SMILES) strings and protein amino acid sequences, graphs, or two-dimensional (2D) representations like predicted contact maps [14–17]. These methods are cost-effective and sufficiently accurate compared to experimental or *in silico* structure prediction [18], as they are independent of the protein’s structure when predicting effects.

Structure-based approaches require three-dimensional (3D) protein structures and 1D or 2D molecular inputs. 3D structures are usually derived from experimental data, although computational predictions are increasingly employed [19–23]. These methods have great potential but can be unreliable. They depend on accurate 3D protein structures and may be limited in their ability to generalise beyond experimentally observed DTIs [24]. Due to the complexity of the experimental setup, 3D protein structures can be difficult to obtain. In addition, models often overlook the fact that proteins are not rigid structures, but are generally in motion, e.g., ligand binding induces a conformational change [20, 22, 23].

Finally, complex-based approaches require protein–ligand co-crystal structures, which additionally require 3D information, as well as protein interaction information about the ligand [25]. For this reason, complex-based approaches can provide a more detailed insight into the interactions, but they are by far the most difficult to obtain data for.

Considering these different approaches, we designed BarlowDTI as a fully data-driven, sequence-based approach that relies on SMILES and amino acid sequences as the most accessible data, avoiding costly and time-consuming experimental data such as crystal structures. Additionally, we use a specialised bilingual protein language model (PLM) to embed the 1D amino acid sequence, which uses a 3D-alignment method that results in a “structure-sequence” representation [26, 27]. This approach makes BarlowDTI input data structure-agnostic, yet benefits from “structure-sequence” PLM embeddings. Unlike most other methods, we have developed a system that uses a hybrid “best of both worlds” machine learning (ML) and deep learning (DL) approach to improve drug-target interactions prediction performance in low data regimes where training data is limited [28, 29]. We have found that DL architectures such as Barlow Twins [30, 31] are excellent at learning representations [29] that can then be used for gradient boosting machine (GBM) training to achieve state-of-the-art performance, as the size of datasets is usually too small to reliably train a DL model that will perform competitively.

**Fig. 1:**
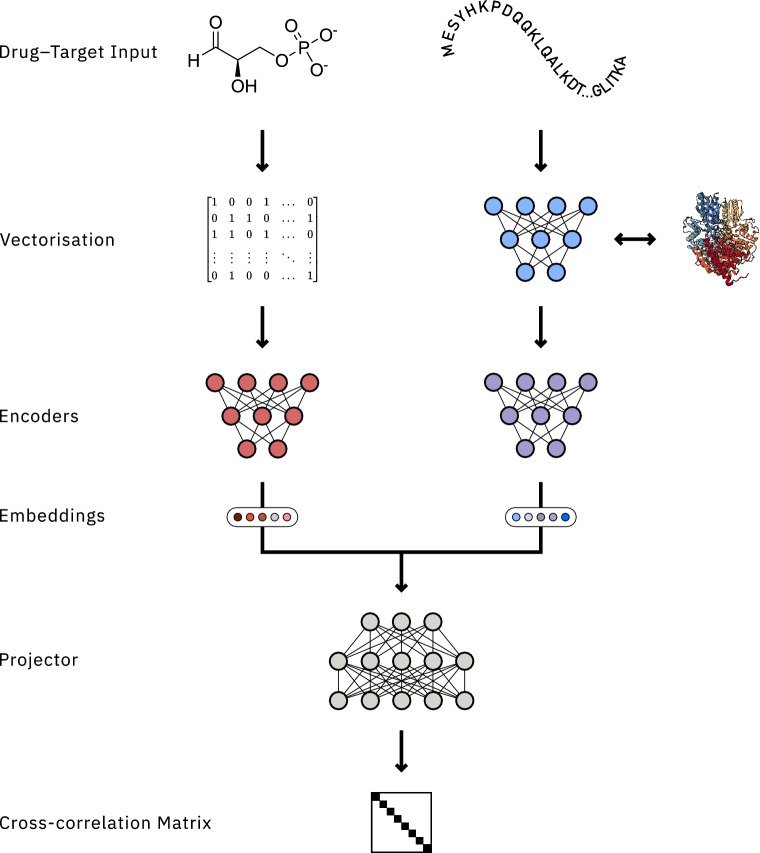
BarlowDTI architecture. Drug and target serve as 1D input, where they are processed and converted into vectors. Molecules are provided as SMILES and converted to ECFP. On the other hand, the primary amino acid sequence is vectorised using a bilingual 3D structure-aware PLM. The Barlow Twins architecture learns to understand drug-target interactions. The objective function forces both representations of the drug-target interactions to be as close as possible to the unity matrix. Finally, this DL model is used as a feature-extractor and a GBM is trained on the embeddings and the interaction label. The GBM is then used as the predictor

To overcome the limitation of data scarcity, we built BarlowDTI_XXL_, which is trained on millions of curated drug-target interactions pairs [32], to apply the model to real-world examples, as we have done in case studies. Here, BarlowDTI_XXL_ captures the correlation between experimentally determined affinities and the predicted likelihood of interaction, proving our approach useful in drug discovery settings. By comparing co-crystal biochemical structures and their active sites, we also investigate and explain how BarlowDTI_XXL_ arrives at its decision. We conduct our investigation by employing an influence method and adapting it in a novel way to identify the most important training DTIs [33]. We also assess whether BarlowDTI_XXL_ can identify ligand binding sites. Additionally, we evaluate its performance in virtual screening experiments, focusing on early detection of hit molecules. This work culminates in a freely available web interface that takes 1D input of molecule and protein information and predicts the likelihood of interaction.

## Results and discussion

### BarlowDTI design

We propose a novel method for predicting DTIs using SMILES notations, primary amino acid sequences, both 1D, and annotated interaction properties. BarlowDTI relies on several key components, visualised in Fig. 1:

1. Firstly, the input needs to be vectorised. We investigate all combinations of several molecular and amino acid representations, and selected the best performing pair of modality representations (Additional file 1: Table S3). This is achieved by converting SMILES into extended-connectivity fingerprint (ECFP). Furthermore, we process amino acid sequences with a PLM that uses both modalities, combining 1D protein sequences and 3D protein structure [26].
2. Secondly, we teach the self-supervised learning (SSL) based Barlow Twins model interaction of molecule and protein [30, 31]. The objective function implements invariance of both representations of one interaction while ensuring non-redundancy of the features [30, 31].
3. Finally, BarlowDTI takes a combination of embeddings generated by the encoders from the Barlow Twins DL model and uses them as features to train a GBM based on the interaction annotations [28]. This approach exploits two key strengths: it uses DL to refine representations, and it leverages the power of machine learning in scenarios with limited data. This is particularly relevant for current drug–target interactions benchmarks/datasets, where only around 50000 annotated pairs are publicly available [34–37]. Consequently, we propose BarlowDTI_XXL_ which is trained on more than 3600000 curated drug-target interactions pairs, additionally sourced from PubChem and ChEMBL [38, 39], to obtain generalisability in real-world scenarios [32].

### Benchmark selection

We selected a comprehensive set of literature-based benchmarks to evaluate the performance of BarlowDTI against several leading methods. The benchmarks considered in this study are derived from several key sources. These sources include biomedical networks [34], the US patent database [35], and data detailing the interactions of 72 kinase inhibitors with 442 kinases, representing over 80 % of the human catalytic protein kinome [36], as well as a collection of binding affinities for the protein–ligand complexes in the Protein Data Bank [40]. These datasets provide DTIs as pairs of molecules and amino acid sequences, each coupled to an interaction annotation.

To ensure a fair comparison, BarlowDTI was retrained across all benchmarks. Finally, we assessed the model’s performance in a binary classification as well as regression setting, where the task is to distinguish between interacting and non-interacting drug–target pairs:

- We compared BarlowDTI with a total of seven established drug-target interactions classification models: the model by Kang et al. (1D structure-agnostic) [41], MolTrans (1D substructure-based) [42], DLM-DTI (1D structure-agnostic) [17], ConPLex (1D structure-agnostic) [43], DrugBAN (2D structure-agnostic) [44], PSICHIC (2D structure-agnostic),[16] and STAMP-DPI (2D structure-based) [45]. For instance, Kang et al. fine-tuned a large language model (LLM) based on amino acid sequences [41]. MolTrans uses an efficient transformer architecture to increase the scalability of the model [42]. DLM-DTI introduced a dual language model approach combined with hint-based learning to improve prediction accuracy [17]. ConPLex leveraged contrastive learning to better understand DTIs [43], while DrugBAN focused on interpretable attention mechanisms that provide insights into the interaction process [44]. PSICHIC utilised physicochemical properties to predict interactions more accurately [16], and STAMP-DPI incorporated structure-aware, multi-modal learning to enhance its predictive capabilities [45]. Overall, we evaluated our architecture against the various model implementations. These models have demonstrated state-of-the-art performance in benchmarks.
- This comparison is performed on a total of four classification datasets with twelve predefined literature-proposed splits: 4 $\times$ BioSNAP [16, 34, 41], 4 $\times$ BindingDB [16, 35, 41], 1 $\times$ DAVIS [36, 41] and 3 $\times$ Human [16, 42]. In addition, regression performance is evaluated on the benchmarks PDBBind v2016 and v2020.[25, 46–48] Our aim is to investigate the behaviour of different methods in diverse splitting scenarios, where a whole dataset is split into model training, validation, and evaluation subsets. These predefined splits help us to assess how well models generalise under challenging evaluation conditions, for example where either the drug or the target has not been seen before, thus providing insight into their real-world applicability. A detailed analysis of all benchmarks can be found in the Supporting Information “Dataset analysis”.
- In addition, we investigated the addition of a more rigorous model baseline. The GBM XGBoost is known to be one of the best models, e.g. in quantitative structure-activity relationship (QSAR) tasks, often outperforming DL-based approaches [49–51].

### BarlowDTI shows state-of-the-art performance in predicting DTIs

**Fig. 2:**
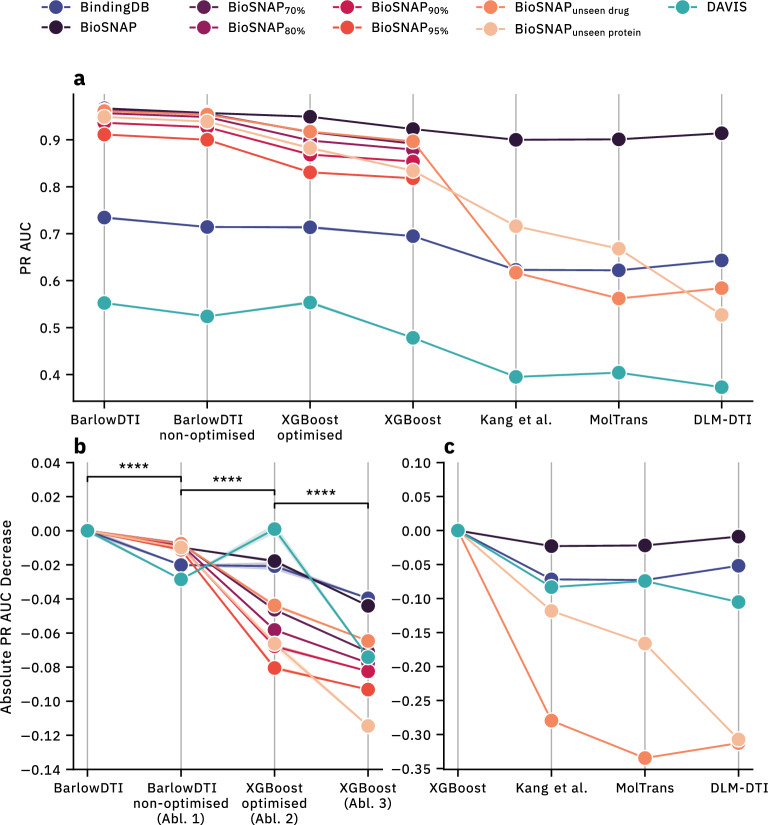
A comparison of the performance of methods established in the literature. **a** The state-of-the-art performance of BarlowDTI in terms of PR AUC was visualised in comparison to other models (for metrics and their statistics refer to Table 1). **b** The change in performance was examined as key elements of the BarlowDTI architecture were incrementally removed. Ablations are denoted as Abl. 1, 2 and 3. **c** The newly introduced model baseline, XGBoost, was compared with other established methods. A per dataset and split difference in PR AUC was calculated based on BarlowDTI in (**b**) performance or the baseline model in (**c**). The overall change was investigated for statistical significance (****$p < 0.0001$, two-sided Welch’s *t*-test [52, 53], with Benjamini-Hochberg [54] multiple testing correction)

 We assessed the performance of BarlowDTI in binary classification across four distinct datasets, each employing different data splitting procedures. For each dataset, we predicted whether drug–target pairs in the predefined test subset interact or not. We then statistically evaluated these predictions by comparing them to the actual outcomes provided in the benchmark test set, using the metrics receiver operating characteristic area under curve (ROC AUC) and precision recall area under curve (PR AUC). Overall, BarlowDTI significantly outperforms all other models in Fig. 2a and Tables 1 and Additional file 1: Table S5. Looking at BioSNAP, we improve 6 % over the leading method DLM-DTI in terms of PR AUC. Furthermore, as shown in Table 2 BarlowDTI again outperforms the PSICHIC method with a 7 % PR AUC improvement independent of the split.

**Table 1:** Benchmarking BarlowDTI against other models using Kang et al. splits [41]

Dataset	Model	ROC AUC	PR AUC
BioSNAP	BarlowDTI	**0.9599** ± **0.0004**	**0.9670** ± **0.0004**
	XGBoost	0.9142	0.9229
	MolTrans [42]	0.895 ± 0.002	0.901 ± 0. 004
	Kang et al. [41]	0.914 ± 0.006	0.900 ± 0.007
	DLM-DTI [17]	0.914 ± 0.003	0.914 ± 0.006
	ConPLex [43]	–	0.897 ± 0.001
BindingDB	BarlowDTI	**0.9364** ± **0.0003**	**0.7344** ± **0.0018**
	XGBoost	0.9261	0.6948
	MolTrans [42]	0.914 ± 0.001	0.622 ± 0.007
	Kang et al. [41]	0.922 ± 0.001	0.623 ± 0.010
	DLM-DTI [17]	0.912 ± 0.004	0.643 ± 0.006
	ConPLex [43]	–	0.628 ± 0.012
DAVIS	BarlowDTI	**0.9480** ± **0.0008**	**0.5524** ± **0.0011**
	XGBoost	0.9285	0.4782
	MolTrans [42]	0.907 ± 0.002	0.404 ± 0.016
	Kang et al. [41]	0.920 ± 0.002	0.395 ± 0.007
	DLM-DTI [17]	0.895 ± 0.003	0.373 ± 0.017
	ConPLex [43]	–	0.458 ± 0.016

Performance was evaluated against three established benchmarks, and the mean and standard deviation of the performance of five replicates are presented. Results per benchmark that are both the best and statistically significant (Two-sided Welch’s *t*-test [52, 53], $\alpha = 0.001$ with Benjamini-Hochberg [54] multiple test correction) are highlighted in bold

When switching to BindingDB, BarlowDTI significantly outperforms DLM-DTI in terms of PR AUC with a >14 % improvement (Table 1). Investigating the BindingDB splits shows that BarlowDTI outperforms all existing methods when looking at unseen ligands, matches the ROC AUC performance of DrugBAN in the random setting and becomes second best in the unseen protein split (Table 2). Overall, BarlowDTI performs best in two out of four splits in this benchmark.

**Table 2:** Benchmarking BarlowDTI against other models using Koh et al. splits [16]

Dataset	Split	Model	ROC AUC	PR AUC
BioSNAP	Unseen protein	BarlowDTI	**0.9572**	**0.9679**
		DrugBAN [16, 44]	0.7327	0.7971
		PSICHIC [16]	0.8819	0.9071
		STAMP-DPI [16, 45]	0.8372	0.8738
		XGBoost	0.8506	0.8794
	Random split	BarlowDTI	**0.9718**	**0.9755**
		DrugBAN [16, 44]	0.9089	0.9159
		PSICHIC [16]	0.9246	0.9226
		STAMP-DPI [16, 45]	0.8993	0.9056
		XGBoost	0.9146	0.9242
	Unseen ligand	BarlowDTI	**0.9666**	**0.9706**
		DrugBAN [16, 44]	0.8775	0.8843
		PSICHIC [16]	0.9019	0.9030
		STAMP-DPI [16, 45]	0.8902	0.8915
		XGBoost	0.8909	0.9026
BindingDB	Unseen protein	BarlowDTI	0.6939	0.5791
		DrugBAN [16, 44]	0.6523	0.5295
		PSICHIC [16]	**0.7537**	**0.6241**
		STAMP-DPI [16, 45]	0.6828	0.5735
		XGBoost	0.6460	0.5233
	Random split	BarlowDTI	**0.9640**	0.9513
		DrugBAN [16, 44]	**0.9640**	**0.9539**
		PSICHIC [16]	0.9503	0.9280
		STAMP-DPI [16, 45]	0.9318	0.9085
		XGBoost	0.9582	0.9462
	Unseen ligand	BarlowDTI	**0.9456**	**0.9263**
		DrugBAN [16, 44]	0.9409	0.9188
		PSICHIC [16]	0.9264	0.8975
		STAMP-DPI [16, 45]	0.9027	0.8683
		XGBoost	0.9374	0.9141
Human	Unseen protein	BarlowDTI	**0.9630**	**0.9693**
		DrugBAN [16, 44]	0.9298	0.9417
		PSICHIC [16]	0.9503	0.9595
		STAMP-DPI [16, 45]	0.8563	0.8748
		XGBoost	0.8961	0.9171
	Random split	BarlowDTI	**0.9917**	**0.9905**
		DrugBAN [16, 44]	0.9841	0.9753
		PSICHIC [16]	0.9861	0.9840
		STAMP-DPI [16, 45]	0.9659	0.9582
		XGBoost	0.9813	0.9782
	Unseen ligand	BarlowDTI	0.9346	0.9348
		DrugBAN [16, 44]	0.9459	**0.9387**
		PSICHIC [16]	**0.9500**	0.9371
		STAMP-DPI [16, 45]	0.9156	0.8980
		XGBoost	0.9391	0.9337

Performance was evaluated against three established benchmarks, and the mean of the BarlowDTI performance of five replicates are presented. All other metrics are taken from Koh et al. Best result per benchmark and split is highlighted in bold. Koh et al. does not present replicates or sample-correlated predictions [16]

BarlowDTI once again outperforms all of the established approaches when looking at the DAVIS benchmark, with a 21 % improvement over the leading ConPLex model in terms of PR AUC (Table 1).

Furthermore, we evaluated the performance on the Human benchmark. BarlowDTI shows the best performance when looking at the unseen protein split as well as the random split (Table 2). PSICHIC comes first in the unseen ligand setting, when looking at ROC AUC, while DrugBAN is best in PR AUC. In summary, BarlowDTI outperforms all other models in two out of three splits.

Lastly, BarlowDTI and XGBoost regression performance was determined on two regression benchmarks: PDBBind v2016 and v2020 (Additional file 1: Tables S4 and S8) [25, 46–48]. Overall, BarlowDTI demonstrates competitive regression performance, ranking third on PDBBind v2016 and second on PDBBind v2020 among twelve literature-known models (Supporting Information “Regression performance”). XGBoost places third and fourth, still putting our proposed baseline ahead of two-thirds of all methods.

We looked at the architecture and its components, removing one at a time and measuring the effect on performance to investigate why BarlowDTI outperforms other methods in various benchmarks.

### Unravelling the performance contributions of the BarlowDTI architecture

 To investigate the impact of each element of the BarlowDTI architecture, we removed them one at a time. We have done this across all baselines and splits with the following ablations:

1. We removed the hyperparameter optimisation step of the BarlowDTI classifier (Fig. 2b Abl. 1).
2. From the first removal, we replaced the Barlow Twins architecture entirely and instead concatenate ECFP and PLM embeddings for training (Fig. 2b Abl. 2). We kept the hyperparameter optimisation procedure as in BarlowDTI.
3. Finally, we removed the hyperparameter optimisation procedure from the previous ablation, analogous to the first modification (Fig. 2b Abl. 3).

We observe a significant decline in performance, as illustrated in Fig. 2b and Additional file 1: Table S6 for the initial ablation, emphasising the crucial role of hyperparameter optimisation for achieving optimal model performance.

The second ablation also indicates a significant reduction in performance. However, for the DAVIS benchmark, the optimised GBM demonstrated surprisingly strong performance. We hypothesise that this may result from the hyperparameter search potentially leading to overfitting on the test set. This would be consistent with the improvement from the third ablation experiment to the first experiment comparing the two non-optimised model variants. On the whole, model performance is likely attributed to the DL architecture based on the SSL Barlow Twins model, which effectively learns embeddings to describe DTIs. The Barlow Twins objective promotes orthogonality between drug and target modalities while ensuring the non-redundancy of both, thus preventing informational collapse. As a result, this leads to an overall state-of-the-art predictive performance.

The final ablation shows a further decline in performance, consistent with the results of the initial ablation experiment.

In summary, the sustained reduction in performance of our ablation experiments demonstrates that each component of our BarlowDTI pipeline is needed to maximise performance. This architecture integrates the “best of both worlds”: DL and GBM to enhance predictive performance. Compared to other pure machine learning- or DL-based approaches, we can demonstrate a performance boost. In particular, the use of a state-of-the-art PLM [26] could offer an advantage over other methods. Other PLM variants are ProtTrans [55] in ConPLex [43] and ProtBERT proposed by Kang et al. also used in DLM-DTI [41]. The structural awareness of BarlowDTI added by the inclusion of 3D-alignment in ProstT5 [26] hints towards better generalisation capabilities, yielding increased performance.

#### Choosing baseline models

Selecting an appropriate baseline model is critical to effectively comparing different machine learning and DL techniques. Robust baselines are the basis for meaningful comparisons and highlight improvements from new methods. Without appropriate baselines, it becomes difficult to determine whether new approaches are truly advancing the field.

Current leading drug-target interactions models predominantly use DL methods and are often evaluated against simple baseline models such as logistic regression, ridge or deep neural network (DNN) classifiers [42, 43]. To improve the benchmarking process, we propose to add GBMs as a baseline for drug-target interactions benchmarking purposes, as shown in the final ablation configuration. GBMs such as XGBoost have demonstrated broad adaptability, e.g. in QSAR modelling, offering strong predictive performance and fast training times, particularly in scenarios with limited data availability, such as drug-target interactions prediction.

We compared the overall model performance across all datasets in Fig. 2c and Tables 1, 2, and Additional file 1: Tables S4, S7, and S8. Here, the performance of XGBoost trained on ECFP and PLM embeddings is highlighted as it shows competitive performance across all methods and datasets.

### Demonstration of the capabilities of BarlowDTI_XXL_

 To use BarlowDTI in real-world applications, more training data is needed to predict meaningful interactions. For this purpose, we have built BarlowDTI_XXL_, which is trained on more than 3600000 curated drug-target interactions pairs [32]. We have kept the same model design to ensure the comparability and performance of our hybrid approach. We looked at several co-crystal structures as case studies to provide insight into the possibilities using BarlowDTI_XXL_. In order to demonstrate the ability to generalise beyond the learnt DTIs, we evaluated our approach on structures which are not part of the training set. Our aim is to demonstrate the applicability of the model to multiple structures and affinities, as in the study performed by Dienemann et al. The importance of this work is further emphasised by its relevance to the malaria-causing parasite *Plasmodium falciparum* [56].

We first analysed the co-crystal structures *Plasmodium falciparum* lipoate protein ligase 1 LipL1 (5T8U) and *Listeria monocytogenes* lplA1 (8CRI), which share a low sequence identity (28.7 %) despite their structural similarity. Our objective is to evaluate the model’s ability to generalise, particularly when only 1D input is provided. This evaluation focuses on the model’s performance in capturing both biological function and structural attributes under these conditions. Secondly, we examined the predictive shifts induced by ligand methylation and explored the interaction dynamics of a novel enzyme inhibitor C3 (8CRL). This case study is further enriched with isothermal titration calorimetry (ITC) data [56], offering insights into the ligand’s affinity towards the target proteins.

**Fig. 3:**
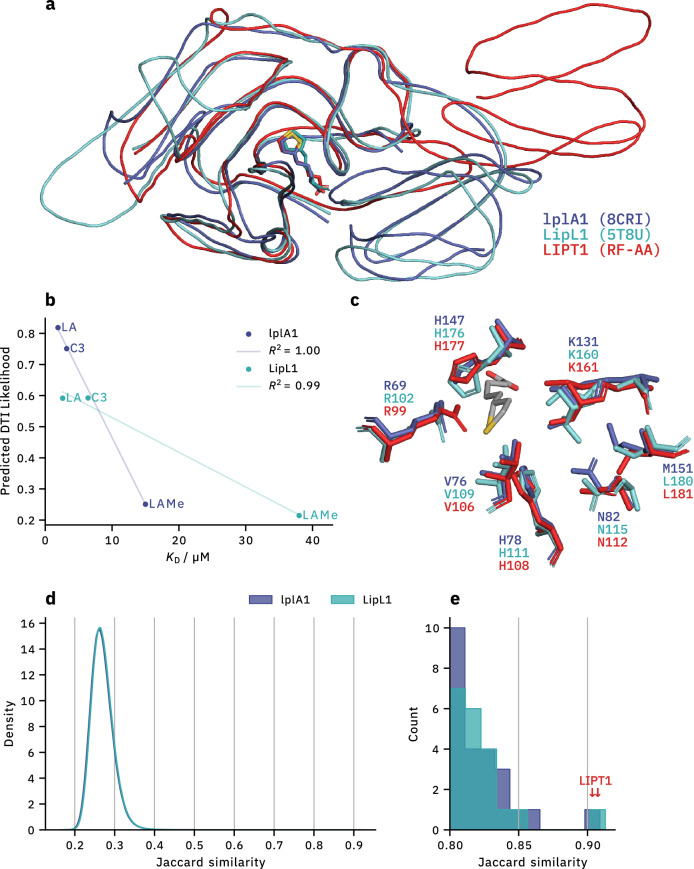
Structure-based explanation of BarlowDTI_XXL_ predictions. **a** Co-crystal structures of lplA1 and LipL1 with LA as ligand are shown in superposition, together with the most influential training sample (structure predicted using RoseTTAFold-AllAtom (RF-AA) [21]). **b** The squared Pearson *R* [60] correlation of BarlowDTI_XXL_ and ITC measurements is presented [56]. **c** The protein residue–ligand interactions at the active site are compared. **d** We identified the most influential training samples for LA predictions. The distribution of Jaccard similarity for all training samples is shown. We applied kernel density estimation to the histogram to improve visibility, due to the large training set size. **e** The most influential training samples are highlighted ($\downarrow$)

Our results indicate, that BarlowDTI_XXL_ is able to accurately predict the correlation between the experimentally determined affinity measured via ITC and the likelihood of the DTI (Fig. 3b). These capabilities provide useful insight in the drug discovery process, as researchers are able to prioritise chemical scaffolds. BarlowDTI_XXL_ is able to catch small changes in the ligands structure and accurately predict the shift in interaction likelihood. This is illustrated by the methylation of LA, where our method predicts a significant decrease in interaction likelihood, consistent with the decrease in affinity measured by ITC.

To further validate the performance of BarlowDTI_XXL_, we conducted a virtual screening experiment (Additional file 1: Fig. S2) focused on identifying kinase inhibitors. Kinase inhibitors are pivotal in drug discovery, targeting dysregulated protein kinases linked to cancer, autoimmune disorders, and inflammation [57–59]. Our results show that BarlowDTI_XXL_ effectively prioritised kinase inhibitors while remaining computationally efficient, reducing costs and time, and thereby accelerating the drug discovery process (Supporting Information “Virtual screening”).

We looked at Shapley additive explanation (SHAP) values to examine the influence of each input modality on the model (Additional file 1: Fig. S6). Regardless of the ligand molecule chosen, each modality proved equally important for prediction. This finding highlights the functionality and predictive power of BarlowDTI’s architecture.

### Explaining BarlowDTI by investigating sample importance

 We analysed the importance of individual samples within the training set to understand how BarlowDTI classifies DTIs. Our adjusted influence method was therefore applied. In Fig. 3d,e, we identified the most influential training pairs by examining those with the highest Jaccard similarity, calculated from the leaf indices of the GBM in BarlowDTI_XXL_. The most influential training sample is the *Homo sapiens* lipoyl amidotransferase LIPT1 for both lplA1 and LipL1, with LA as the common ligand (Fig. 3a,e). LIPT1 and lplA1 ($J = 0.909$) share a sequence identity of 31.8 %, while LIPT1 and LipL1 ($J = 0.913$) only share 29.7 % (Fig. S7).

To investigate the biochemical implications of the training sample to the model’s prediction, we performed a structural study. We leveraged protein crystal structures to perform in-depth 3D analyses on lplA1 (8CRI) and LipL1 (5T8U). The superposition of lplA1 with LIPT1 revealed a root mean square deviation of atomic positions (RMSD) of 2.07 Å, while LipL1 exhibited a RMSD of 1.72 Å. These RMSD values reflect a significant structural congruence among these enzymes, notwithstanding their low sequence identity. Despite this structural similarity, it is noteworthy that human LIPT1 does not catalyse the same reaction as lplA1 and LipL1 [61].

Furthermore, we looked at the active site of LipL1, where all residues are conserved relative to LIPT1 (Fig. 3c). In lplA1, one notable substitution can be observed. L181 in LIPT1 is replaced by M151, possibly explaining the higher Jaccard similarity of LipL1 over lplA1. This conservation pattern underscores a highly conserved binding pocket across species, as confirmed by sequence alignment data (Additional file 1: Fig. S7). Furthermore, we investigated whether BarlowDTI_XXL_ demonstrates sequence-based awareness of ligand interaction sites. The strongest shifts in predicted drug–target interaction likelihood is observed when active site residues are substituted (Additional file 1: Figs. S3 and S4, Supporting Information “Ligand interaction”). These results highlight the awareness of BarlowDTI_XXL_ to ligand-binding residues and help to understand how the prediction of the model is achieved.

In summary, BarlowDTI_XXL_ effectively learns DTIs by leveraging catalytically active and stabilising residues, demonstrating the model’s ability to generalise from 1D input data. This capability makes BarlowDTI_XXL_ well-suited for applications in drug discovery.

## Conclusions

Our proposed method, BarlowDTI, integrates sequence information with the Barlow Twins SSL architecture and GBM models, representing a powerful fusion of machine learning and DL techniques.

Our approach demonstrates state-of-the-art drug-target interactions prediction capabilities, validated across multiple benchmarks and data splits. Notably, our method outperforms existing literature benchmarks in ten out of fourteen datasets evaluated.

To elucidate the efficacy of BarlowDTI, we conducted an ablation study to investigate the contribution of its core components and their impact on performance. In addition, we re-evaluated the choice of baselines in numerous publications and advocate the inclusion of GBM baselines. Furthermore, we explored the classification mechanism of BarlowDTI for DTIs by performing a structure-based analysis of the most influential training samples. This was done by adapting a previously developed influence method to gain deeper insight into training sample importance.

Given the model’s exceptional performance, we are confident that BarlowDTI can significantly accelerate the drug discovery process and offer significant time and cost savings through the use of virtual screening campaigns. To make BarlowDTI accessible to the scientific community, we provide an easy-to-use and free web interface at https://www.bio.nat.tum.de/oc2/barlowdti.

## Methods

### Datasets

To evaluate the performance of BarlowDTI, three established benchmarks are used. They all provide fixed splits for training, evaluation and testing. In some publications the training and evaluation is merged to improve predictive performance. To ensure comparability, this was not done in this work. All the metrics presented are taken from other publications in which only the training set is used.

In addition, Kang et al. first proposed splits for large drug-target interactions datasets, BioSNAP [34], BindingDB [35] and DAVIS [36, 41].

The addition of a variety of splits with an additional benchmark Human [42] are proposed by Koh et al. We evaluate these separately [16]. Regression performance is evaluated on two regression benchmarks: PDBBind v2016 and v2020 [25, 46–48]. For all datasets, to reduce bias and improve model performance, the SMILES are cleaned using the Python ChEMBL curation pipeline [62]. All duplicate and erroneous molecule and protein information that could not be parsed is removed. Training is performed on the predefined training splits.

### Representations

#### Molecular information

 The SMILES are converted into Atom Pair [63], ECFP [64], Electrotopological State (EState) [65], MACCS [66], MinHashed Atom Pair (MAP) [67], PubChem and RDKit fingerprints [68] using scikit-fingerprints and RDKit [68, 69]. We used 1024bit and a radius of 2 where possible, otherwise the default parameters were used.

#### Amino acid sequence information

 The amino acid sequences are converted into vectors, by using the PLM ProtTrans [55], ProtT5 [55] and ProstT5 [26]

Additionally, the protein sequences were encoded using one-hot encoding, In this method, each amino acid is represented by a unique binary vector where one position is set to 1, and all others are set to 0. For this encoding, we used the standard set of 20 amino acids $\mathcal {A}$: Each amino acid $a \in \mathcal {A}$ is mapped to a unique index: $\text {index}(a) = i, \quad \text {where} \, i \in \{0, 1, \dots , 19\}.$ For a given amino acid sequence $S = (s_1, s_2, \dots , s_n)$, where each $s_j \in \mathcal {A}$, we encode each amino acid $s_j$ as a one-hot vector $\textbf{v}_j \in \mathbb {R}^{20}$, defined as:

$$\textbf{v}_j[i] = {\left\{ \begin{array}{ll} 1 & \text {if } i = \text {index}(s_j),\\ 0 & \text {otherwise}. \end{array}\right. }$$

For sequences shorter than a predefined maximum length $l_{\max }$, padding is applied using a placeholder amino acid $\text {X}$, which is mapped to a zero vector: $\textbf{v}_{\text {X}} = (0, 0, \dots , 0) \in \mathbb {R}^{20}.$ Given a sequence of length $n$, we ensure the final encoded vector has length $20 \times l_{\max }$ by either truncating or padding the sequence. Thus, for each sequence $S$ of length $n$, the one-hot encoded representation $\textbf{V}(S)$ is given by:

$$\textbf{V}(S) = [ \textbf{v}_1, \textbf{v}_2, \dots , \textbf{v}_{l_{\max }} ] \in \mathbb {R}^{20 \times l_{\max }}.$$

### Barlow Twins model configuration

The proposed method is based on the Barlow Twins [30] network architecture, which employs one encoder for each modality and a unified projector. The encoders and projector are multilayer perceptron (MLP) based.

Both encoders as well as the projector have the following structure

$$\begin{aligned} \varvec{l_{i+1}} = \textrm{Linear} \left( {\textrm{ReLU} \left( \textrm{BatchNorm} \left( \textrm{Linear} \left( \varvec{W l_i + b} \right) \right) \right) }^{n} \right) , \end{aligned}$$

where $\varvec{l_{i}}$ is the input layer and $\varvec{l_{i+1}}$ is its output, with a flexible number of layers *n* and adjustable dimensionality of input and output. Furthermore, variables $\varvec{W}$, $\varvec{b}$ represent learnable weights and biases. A linear layer is followed by batch normalisation [70], ReLU activation function [71], and the last linear layer. The network was constructed using PyTorch [72].

The loss function $\mathcal {L_{BT}}$ is adapted from the original Barlow Twins publication and enforces cross-correlation (matrix $\mathcal {C}$) between the projections of the modalities [30].

$$\begin{aligned} \mathcal {L_{BT}} \triangleq \underbrace{\sum _i (1-\mathcal {C}_{ii})^2}_\text {invariance term} + ~~\lambda \underbrace{\sum _{i}\sum _{j \ne i} {\mathcal {C}_{ij}}^2}_\text {redundancy reduction term} \end{aligned}$$

where $\lambda$ is a constant that trades off the invariance term and redundancy reduction term.

$$\begin{aligned} \mathcal {C}_{ij} \triangleq \frac{ \sum _b z^A_{b,i} z^B_{b,j}}{\sqrt{\sum _b {(z^A_{b,i})}^2} \sqrt{\sum _b {(z^B_{b,j})}^2}} \end{aligned}$$

#### Pre-training Barlow Twins

Here we pre-train the Barlow Twins architecture on our joint drug-target interactions dataset, based on BioSNAP, BindingDB, DAVIS (Kang et al. splits) and DrugBank [37], removing duplicates and without labels to teach DTIs. Early stopping is implemented to avoid overfitting, which is carried out using a 15 % validation split.

##### Hyperparameter optimisation

Manual hyperparameter optimisation is performed, shown in Table 3.

**Table 3:** Barlow Twins hyperparameters

Hyperparameter	Value/Range
enc_n_neurons	1024, 2048, **4096**
enc_n_layers	1, 2, **3**
proj_n_neurons	1024, **2048**, 4096
proj_n_layers	**1**, 2, 3
embedding_dim	**512**, 1024, 2048
act_function	ReLU
aa_emb_size	1024
loss_weight	1 $\times 10^{-5}$, **0.005**, 0.1
batch_size	4096
epochs	250
optimizer	AdamW
learning_rate	1 $\times 10^{-5}$, **3** $\times 10^{-4}$, 0.1
beta_1	0.9
beta_2	0.999
weight_decay	5$\times 10^{-5}$
step_size	10
gamma	0.1
val_split	0.1

The best values are marked in bold

#### Feature-extractor

When performing feature-extraction, we use the pre-trained BarlowDTI model. For training and prediction, we extract the embeddings after the encoders for each modality and concatenate them. Finally, a GBM, XGBoost [28] Python implementation, is trained on the embeddings in combination with the labels for each training sets respectively.

#### *Hyperparameter optimisation*

If a benchmark provides a dedicated validation set, this was used for Optuna [73] hyperparameter optimisation. Therefore, in classification $\mathcal {L}_{c} = \text{ROC AUC} + \text{PR AUC}$ was used as validation loss and Optuna was configured to maximise the summed loss. For regression $\mathcal {L}_{r} = - \rho + \text{MAE}$ was applied as validation loss and Optuna was configured to minimise the summed loss.

The optimisation was carried out for 100 trials with the parameters shown in Table 4. The obtained benchmark specific hyperparameters were then used to fit the GBM on the training set. All detailed hyperparameters are provided in the Additional File gbm_hyperparameters.csv.

**Table 4:** GBM hyperparameters

Hyperparameter	Value/Range
n_estimators	[100, 1000] (step = 100)
learning_rate	[1e−8, 1.0] (log scale)
max_depth	[2, 12]
gamma	[1e−8, 1.0] (log scale)
min_child_weight	[1e−8, 1e2] (log scale)
subsample	[0.4, 1.0]
reg_lambda	[1e−6, 10] (log scale)

Best parameters differ for each benchmarking dataset and split

##### **BarlowDTI**_**XXL**_

We introduce BarlowDTI_XXL_, a model trained for use in real-world applications. To build BarlowDTI_XXL_, we curated and standardised the large drug-target interactions dataset proposed by Golts et al. (procedure adapted from the “Datasets” section) [32]. Furthermore, we used random undersampling with a 3:1 ratio of non-interactors to interactors to improve model generalisation. Then we added the training splits from BioSNAP, BindingDB and DAVIS (Kang et al. splits), resulting in a model trained with 3653631 drug-target interactions pairs (2 789 498 non-interactors, 864 133 interactors).

BarlowDTI_XXL_ uses the same architecture as BarlowDTI, using the powerful Barlow Twins network as feature-extraction method in combination with the GBM XGBoost [28, 30].

##### **Baseline model configuration**

As a baseline, we have selected a GBM. Similar to our feature-extraction implementation, for all features we concatenate both ECFP and PLM embeddings. Finally, a GBM, XGBoost Python implementation, is trained on the ECFP and PLM embedding concatenation in combination with the labels for each training set, respectively.

##### **Case study**

Amino acid sequence information as well as ligand information is taken from The Protein Data Bank to perform predictions using BarlowDTI [74]. Complex structures were generated using RoseTTAFold-AllAtom (RF-AA) [21].

Sequence identity was determined. Therefore, sequences were aligned using the BLASTP [75, 76] algorithm at https://blast.ncbi.nlm.nih.gov [77]. PyMOL 2 is used for structure visualisation and RMSD value calculation [78].

###### ***Explainability based on Shapley additive explanation values***

We applied the TreeExplainer [79, 80] algorithm to the GBM of BarlowDTI_XXL_ extracted and visualised the SHAP values.

###### ***Explainability based on sample importance***

To assess how the model decides to classify drug–target pairs as interacting or non-interacting, we looked at the influence of training samples, as similarly proposed by Brophy et al. for uncertainty estimation [33]. We used a similar concept but changed the approach to identify the most influential training data. This is done by obtaining the leaf indices of the GBM of all training samples. Then we compare the leaf indices at inference time with the leaf indices of the training samples. Finally, we find the most influential samples by computing the pairwise Jaccard similarity of the leaf index vectors [81],

$$\begin{aligned} J(A, B)=\frac{|A \cap B|}{|A \cup B|}. \end{aligned}$$

The most influential training sample is represented by the maximum Jaccard similarity.

## Supplementary Information

Supplementary Material 1. Supporting File 1.

## Data Availability

The easy-to-use web interface can be found at https://www.bio.nat.tum.de/oc2/barlowdti. The code is available on GitHub https://github.com/maxischuh/BarlowDTI. Also available on GitHub are the curated and extensive BarlowDTI_XXL _training, as well as the benchmark data. The system used for computational work is equipped with an AMD Ryzen Threadripper PRO 5995WX CPU with 64/128 cores/threads and 1024GB RAM. The server is also powered by an NVIDIA RTX 4090 GPU with 24GB VRAM.

## Abbreviations

1D: One-dimensional
2D: Two-dimensional
3D: Three-dimensional
BEDROC: Boltzmann-enhanced discrimination of receiver operating characteristic
CI: Concordance index
DL: Deep learning
DNN: Deep neural network
DTI: Drug-target interaction
ECFP: Extended-connectivity fingerprint
GBM: Gradient boosting machine
ITC: Isothermal titration calorimetry
LA: Lipoic acid
LLM: Large language model
MAE: Mean absolute error
ML: Machine learning
MLP: Multilayer perceptron
PLM: Protein language model
PR AUC: Precision recall area under curve
QSAR: Quantitative structure-activity relationship
RF-AA: RoseTTAFold-AllAtom
RMSD: Root mean square deviation of atomic positions
ROC AUC: Receiver operating characteristic area under curve
SHAP: Shapley additive explanation
SMILES: Simplified molecular-input line-entry system
SSL: Self-supervised learning
