# Supplementary material for: Barlow Twins deep neural network for advanced 1D drug–target interaction prediction
Source: J Cheminform. 2025 Feb 5;17:18. doi: 10.1186/s13321-025-00952-2 (PMC11800607; doi:10.1186/s13321-025-00952-2)
Supplement: Supplementary file 1 — Supplementary Material 1. Supporting File 1. [file 13321_2025_952_MOESM1_ESM.pdf]

Supporting Information – Barlow Twins deep neural network for advanced 1D drug–target interaction prediction

Maximilian G. Schuh[1], Davide Boldini[1*], Annkathrin I. Bohne[2], Stephan A. Sieber[1*]

[1*]TUM School of Natural Sciences, Department of Bioscience, Center for Functional Protein Assemblies (CPA), Chair of Organic Chemistry II, Technical University of Munich, Ernst-Otto-Fischer Str. 8, Garching bei München, 85748, Bavaria, Germany.

[2]TUM School of Natural Sciences, Department of Bioscience, Center for Functional Protein Assemblies (CPA), Chair of Biochemistry, Technical University of Munich, Ernst-Otto-Fischer Str. 8, Garching bei München, 85748, Bavaria, Germany.

*Corresponding author(s). E-mail(s): davide.boldini@tum.de; stephan.sieber@tum.de;

Additional Results and Discussion

Dataset analysis

To better understand the benchmarks used in this study, we analysed the number of drug–target interaction (DTI) pairs, unique drugs and targets, and the ratio of positive interactions or mean label $\bar{y}$ per split (Tabs. S1 and S2). All benchmarks are non-sparse, typically containing data of drug molecules tested against multiple targets. We generated interaction maps for each training set (Fig. S1). Due to the high dimensionality of the axes, zooming in may be necessary to observe interactions in detail. The interactions are summed, with darker colours indicating a higher number of interactions between modalities. In summary, we observe a relatively smooth distribution of DTI interactions, with only a few dark horizontal and vertical lines indicating a potential dataset bias toward specific drug and target entities.

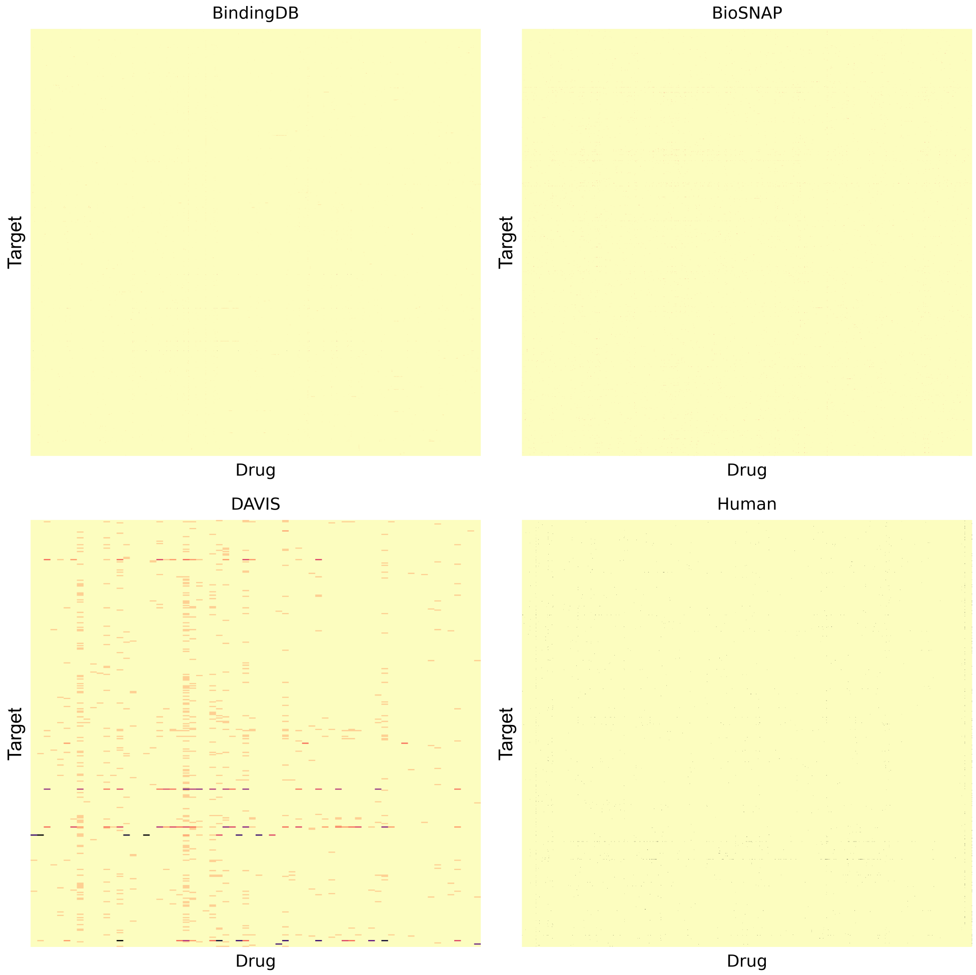

Fig. S1: Training set interaction map of drug and target entities. Zooming in may be necessary to observe interactions in detail. Darker colours indicate a higher number of interactions between modalities.

Tab. S1: Benchmark statistics of BioSNAP, BindingDB, and DAVIS benchmarks using Kang *et al.* splits.[1–4]

**Dataset**	**Split**	**Number of Pairs**	**Unique Drugs**	**Unique Targets**	$\bar{y}$
BindingDB	Training	12 667	3811	1025	0.500
	Validation	6642	1790	812	0.140
	Testing	13 284	3313	980	0.143
BioSNAP full data	Training	19 238	4391	2176	0.503
	Validation	2748	1874	1354	0.508
	Testing	5497	2958	1811	0.504
BioSNAP missing data 70	Training	8245	3555	2019	0.504
	Validation	1924	1454	1099	0.497
	Testing	17 314	4333	2171	0.504
BioSNAP missing data 80	Training	5495	2942	1821	0.498
	Validation	2198	1601	1162	0.515
	Testing	19 790	4414	2179	0.504
BioSNAP missing data 90	Training	2748	1951	1325	0.504
	Validation	2474	1775	1238	0.512
	Testing	22 261	4453	2181	0.502
BioSNAP missing data 95	Training	1376	1145	860	0.495
	Validation	2612	1823	1284	0.517
	Testing	23 495	4468	2180	0.502
BioSNAP unseen drug	Training	19 154	3583	2177	0.498
	Validation	2736	1774	1347	0.505
	Testing	5593	902	1795	0.522
BioSNAP unseen protein	Training	19 375	4374	1744	0.510
	Validation	2768	1893	1201	0.499
	Testing	5340	2927	436	0.483
DAVIS	Training	2086	68	372	0.500
	Validation	3006	68	378	0.053
	Testing	6011	68	379	0.050

Tab. S2: Benchmark statistics of BioSNAP, BindingDB, Human, PDBBind v2016 and v2020 benchmarks using Koh *et al.* splits.[1–3, 5–9]

Dataset	Split	Number of Pairs	Unique Drugs	Unique Targets	$\bar{y}$
BindingDB unseen protein	Training	34 236	13 622	1836	0.413
	Validation	3907	3240	261	0.534
	Testing	11 054	7772	525	0.403
BindingDB random	Training	34 437	13 870	2346	0.423
	Validation	4920	4047	1083	0.410
	Testing	9840	6942	1441	0.414
BindingDB unseen ligand	Training	34 437	10 068	2333	0.419
	Validation	4918	1577	800	0.448
	Testing	9842	2982	1382	0.412
BioSNAP unseen protein	Training	18 828	4371	1525	0.494
	Validation	2554	1872	217	0.447
	Testing	6058	3046	436	0.559
BioSNAP random split	Training	19 210	4396	2170	0.504
	Validation	2743	1906	1306	0.509
	Testing	5487	2906	1819	0.500
BioSNAP unseen ligand	Training	19 207	3125	2174	0.510
	Validation	2745	467	1339	0.490
	Testing	5488	907	1747	0.491
Human unseen protein	Training	4022	2041	1401	0.411
	Validation	600	449	200	0.422
	Testing	1375	1003	400	0.529
Human random split	Training	4197	2152	1719	0.440
	Validation	600	465	474	0.418
	Testing	1200	840	777	0.447
Human unseen ligand	Training	4197	1692	1784	0.440
	Validation	599	329	401	0.417
	Testing	1201	705	718	0.445
PDBBind v2016	Training	3390	2721	2243	6.415
	Validation	377	364	318	6.184
	Testing	290	290	221	6.450
PDBBind v2020	Training	17 797	14 112	11 861	6.360
	Validation	968	800	857	6.350
	Testing	363	343	277	6.492

Representation selection

We identified the best performing combination of molecular and amino acid representations to identify the best starting point for BarlowDTI training. We used the BindingDB benchmark as its the largest and most diverse out of the literate-established benchmarks. The molecules were converted into following representations: Atom Pair,[10] extended-connectivity fingerprint (ECFP),[11] Electrotopological State (EState),[12] MACCS,[13] MinHashed Atom Pair (MAP),[14] PubChem and RDKit fingerprints.[15] On the other hand, the protein sequences were encoded using one-hot encoding, and various protein language models (PLMs): ProtTrans,[16] ProtT5[16] and ProstT5.[17] One modality has also been left out for classifier training (marked as None) (Tab. S3).

Finally, we trained one XGBoost classifier per combination. We could observe that ECFPs in combination with ProstT5 provide the best classification performance when looking at the top 3 performing representation pairs for each metric. Therefore, this combination was chosen for BarlowDTI training.

Tab. S3: The gradient boosting machine (GBM) performance was evaluated on all combinations of molecular and amino acid representations. We evaluated on the BindingDB benchmark on the Kang *et al.* splits.[2, 4] The top 3 performing combinations are marked in italics.

ROC AUC	None	One-hot	ProstT5	ProtTrans	ProtT5
**None**	—	0.8318	0.8323	0.8323	0.8333
**Atom Pair**	0.8206	0.9141	*0.9272*	0.9228	0.9260
**ECFP**	0.8251	0.9136	*0.9276*	0.9212	0.9244
**EState**	0.8202	0.9126	0.9214	0.9187	0.9208
**MACCS**	0.8262	0.9080	0.9209	0.9166	0.9193
**MAP**	0.7812	0.8964	0.9116	0.9051	0.9115
**PubChem**	0.8273	0.9102	*0.9262*	0.9205	0.9229
**RDKit**	0.8252	0.9142	0.9255	0.9212	0.9258

PR AUC	None	One-hot	ProstT5	ProtTrans	ProtT5
**None**	—	0.4929	0.4748	0.4793	0.4767
**Atom Pair**	0.5311	0.6574	0.6884	0.6781	*0.6973*
**ECFP**	0.5421	0.6533	*0.6934*	0.6795	0.6826
**EState**	0.5169	0.6522	0.6656	0.6667	0.6670
**MACCS**	0.5407	0.6435	0.6694	0.6538	0.6722
**MAP**	0.4250	0.5958	0.6334	0.6162	0.6272
**PubChem**	0.5376	0.6532	0.6862	0.6742	0.6790
**RDKit**	0.5493	0.6690	*0.6976*	0.6869	0.6930

Regression performance

In addition to classification performance, we evaluated BarlowDTI on two regression benchmarks: PDBBind v2016 and v2020 (Tabs. S4 and S8).[5–8] Overall, BarlowDTI demonstrated competitive performance, ranking third on PDBBind v2016

and second on PDBBind v2020 among twelve models. Our suggested baseline XGBoost also performs well, coming in fourth and third respectively. While PSICHIC achieved the best results, it is computationally more expensive and requires longer calculation times. The difference in performance between BarlowDTI and PSICHIC may be due to their respective pretraining strategies. Unlike PSICHIC, BarlowDTI is not pre-trained on the PDBBind training data due to its continuous regression labels. Instead, BarlowDTI can leverage these labels during pretraining to distinguish between interacting and non-interacting DTIs, which likely positively contributes to its classification performance.

Tab. S4: Regression performance comparison of different methods on PDBBind v2016 and v2020. The top 3 ranked methods are marked in italics. Values are taken from Koh *et al.*.[9]

**Model**	**PDBBind v2016**			
	MAE ↓	ρ ↑	CI ↑	Mean Rank
BarlowDTI	1.174 ± 0.007	0.764 ± 0.006	0.779 ± 0.003	*3.0*
XGBoost	1.139	0.742	0.771	4.0
SMINA[18]	1.673 ± 0.011	0.609 ± 0.008	0.710 ± 0.003	12.0
GNINA[19]	1.232 ± 0.015	0.728 ± 0.009	0.771 ± 0.003	5.7
dMaSIF[20]	1.205 ± 0.037	0.720 ± 0.013	0.760 ± 0.007	6.0
GraphDTA[21]	1.208 ± 0.028	0.698 ± 0.016	0.759 ± 0.005	7.4
TransCPI[7]	1.243 ± 0.034	0.691 ± 0.022	0.741 ± 0.007	9.0
MolTrans[22]	1.264 ± 0.022	0.675 ± 0.019	0.740 ± 0.008	10.0
DrugBAN[23]	1.109 ± 0.005	0.762 ± 0.005	0.784 ± 0.003	*2.4*
WGNN-DTA[24]	1.332 ± 0.017	0.637 ± 0.014	0.721 ± 0.007	11.0
STAMP-DPI[25]	1.197 ± 0.045	0.699 ± 0.020	0.747 ± 0.004	6.7
PSICHIC[9]	1.040 ± 0.029	0.792 ± 0.011	0.795 ± 0.006	*1.0*
	**PDBBind v2020**			
	MAE ↓	ρ ↑	CI ↑	Mean Rank
BarlowDTI	1.066 ± 0.017	0.686 ± 0.009	0.735 ± 0.007	*2.4*
XGBoost	1.095	0.680	0.728	*3.4*
SMINA[18]	1.161 ± 0.007	0.665 ± 0.005	0.740 ± 0.008	4.0
GNINA[19]	1.413 ± 0.015	0.495 ± 0.011	0.674 ± 0.004	11.7
dMaSIF[20]	1.136 ± 0.031	0.629 ± 0.018	0.710 ± 0.017	6.0
GraphDTA[21]	1.223 ± 0.066	0.612 ± 0.016	0.703 ± 0.019	8.7
TransCPI[7]	1.201 ± 0.037	0.604 ± 0.024	0.677 ± 0.011	9.7
MolTrans[22]	1.271 ± 0.051	0.539 ± 0.057	0.666 ± 0.020	11.3
DrugBAN[23]	1.159 ± 0.043	0.657 ± 0.018	0.720 ± 0.011	5.0
WGNN-DTA[24]	1.196 ± 0.055	0.605 ± 0.025	0.697 ± 0.010	8.7
STAMP-DPI[25]	1.176 ± 0.067	0.653 ± 0.028	0.719 ± 0.011	6.4
PSICHIC[9]	1.015 ± 0.031	0.710 ± 0.027	0.751 ± 0.009	*1.0*

Virtual screening

We used PSICHIC,[9] MolTrans,[22] DLM-DTI,[26] and BarlowDTI$_{\text{XXL}}$ to perform a virtual screening, demonstrating the applicability of DTI predictions. To reduce bias for this analysis, we selected the PubChem bioassay 720504 by searching for "kinase

inhibitor" and sorting by the number of active substances, selecting the top assay (Date and time: 18. December 2024, 13:19 GMT+1).[27] This assay focuses on inhibitors of polo-like kinase 1 (Plk1), a conserved serine/threonine protein kinase subfamily crucial for cell proliferation. Plk1 is essential for the survival of cancer cells with activated Ras or inactivated p53 mutations, while normal cells remain unaffected.[28, 29] Additionally, down-regulation of Plk1 effectively triggers apoptosis in cancer cells and suppresses tumor growth in mouse xenograft models. This makes Plk1 an attractive target for anti-cancer therapy. In summary, the corresponding bioassay contains approximately 365 000 molecules, highlighting both the challenges and potential of DTI predictions.

We evaluated virtual screening performance using two metrics: recall as a function of the screened fraction (Fig. S2) and Boltzmann-enhanced discrimination of receiver operating characteristic (BEDROC). BarlowDTI$_{XXL}$ achieves a BEDROC of 0.300, significantly outperforming the random baseline of 0.058. At the top 20 % of predictions, BarlowDTI$_{XXL}$ prioritises actives, capturing 51.4 % of all actives. This result underscores its potential to significantly reduce both time and cost in virtual screening. In contrast, PSICHIC, MolTrans, and DLM-DTI achieve BEDROC scores of 0.072, 0.062, and 0.063, respectively. While three models perform only slightly above random, BarlowDTI$_{XXL}$ demonstrates superior early detection capabilities. These performance discrepancies may stem from differences in training data, availability of model weights, and pipeline design. Additionally, some methods lack detailed documentation outside of benchmarking scenarios, suggesting limitations in out-of-domain generalisation. For all methods, default model weights were used; if unavailable, DAVIS was selected as it is a kinase dataset expected to be in-domain for the bioassay selection.

Conventional docking methods may outperform some deep learning (DL) models, as discussed by Jain *et al.*,[30] but there are two major requirements: substantial computation time and a protein crystal structure with a known binding site. Tools like AlphaFold3[31] and RoseTTAFold-AllAtom (RF-AA)[32] address the need for three-dimensional (3D) structural information but still involve longer per-molecule calculation times. For instance, the AlphaFold3 GitHub repository reports an inference time of 65 s/mol,[31] amounting to 275 d for all 365 000 molecules in the selected assay. In comparison, PSICHIC required 10 h for the same task. In contrast, BarlowDTI$_{XXL}$ achieves the best performance among the models evaluated while completing predictions for all 365 000 DTIs in just 5 min. MolTrans and DLM-DTI demonstrated similar inference times. In summary, BarlowDTI$_{XXL}$ demonstrates real-world applicability through its ability to generalise and prioritise hits, thereby accelerating drug discovery tasks.

Ligand interaction

We investigated ligand interaction interpretability by performing an alanine scan across the lplA1 sequence to evaluate changes in BarlowDTI$_{XXL}$ prediction likelihood when the sequence window was replaced with alanines. The resulting likelihood shifts are plotted against the lipoic acid (LA) distance of each residue in the crystal structure 8CRI. As shown in Fig. S3, a decrease in DTI likelihood is observed for residues within the 110 to 160 range, marked in grey. This region corresponds to the active site and the known binding pocket of LA. The experiment was repeated using the C3

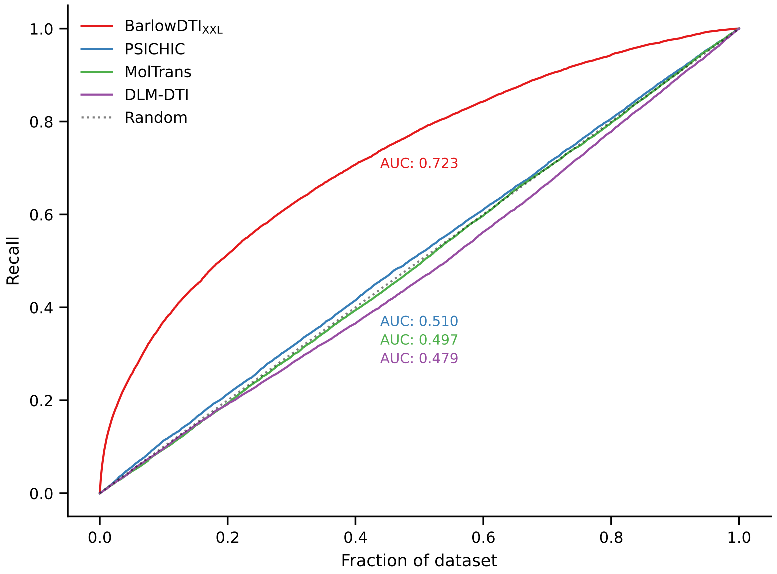

Fig. S2: Recall curve of different methods of bioassay 720504 as a function of the screened fraction. All predictions were ranked by method, and the proportion of actives identified relative to the total number of actives is reported. The area under curve of the recall is presented in the colour of the model.

inhibitor and its corresponding crystal structure 8CRL (Fig. S4). A similar trend is observed, with the largest decrease in likelihood aligning with the inhibitor's binding site, marked in grey, as confirmed by crystallographic data. Overall, this indicates that BarlowDTI$_{XXL}$ shows awareness of the ligand binding site.

The explainability of the ligand binding site is limited by the complexity of the BarlowDTI architecture. Further explaining the model would require addressing three layers and components sequentially: the PLM and simplified molecular-input line-entry system (SMILES) vectorisation, the Barlow Twins network, and the gradient boosting machine (GBM). Deeper explanation is beyond the scope of this work and is left for future investigation.

Statistical testing

We focus on precision recall area under curve (PR AUC) as our classification metric because it is an established performance indicator in unbalanced scenarios. Secondly, it shows a more pronounced separation between different methods, as most methods show very high values of receiver operating characteristic area under curve (ROC AUC). For regression tasks we employ three metrics and use their mean rank as overall performance indicator: mean absolute error (MAE), ρ, and concordance index (CI).

We apply the two-sided Welch's t-test,[33, 34] with Benjamini-Hochberg[35] multiple test correction. This is done for all methods for which the required performance information exists in the published literature.

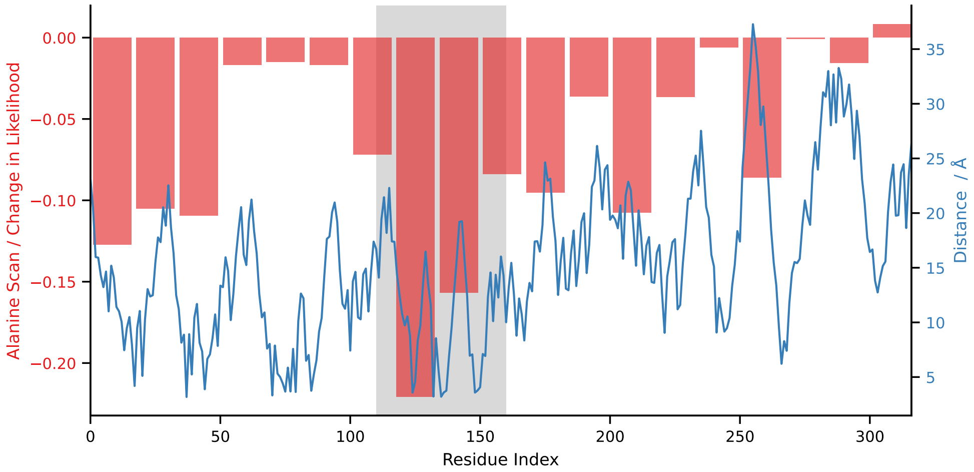

Fig. S3: Alanine scan of lplA1 sequence with LA. The BarlowDTI$_{\mathrm{XXL}}$ prediction likelihood when residues are replaced with alanines is plotted against the LA distance of each residue in the crystal structure 8CRI. The window and binning size used was 20 amino acids.

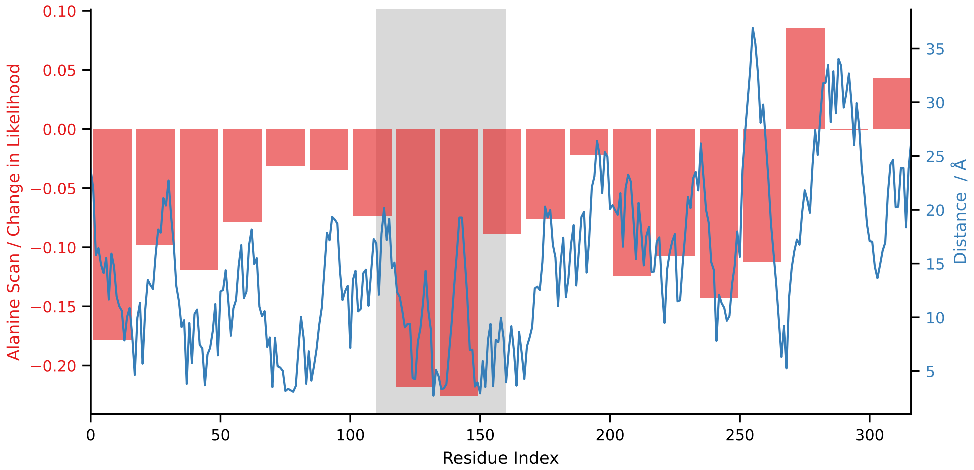

Fig. S4: Alanine scan of lplA1 sequence with C3. The BarlowDTI$_{\mathrm{XXL}}$ prediction likelihood when residues are replaced with alanines is plotted against the C3 inhibitor distance of each residue in the crystal structure 8CRL. The window and binning size used was 20 amino acids.

In Fig. 2, our primary focus is on the overall change in performance. We therefore make comparisons across all datasets collectively rather than individually. Detailed individual comparisons are provided in Tabs. 1 and 2.

Tab. S5: Statistical testing of benchmarking BarlowDTI against other models using Kang *et al.* splits.[4] Five replicates were performed. Two-sided Welch's t-test,[33, 34] $\alpha = 0.001$ with Benjamini-Hochberg[35] multiple test correction was applied.

**Dataset**	**Model**	**ROC AUC**		**PR AUC**	
		p_{corr} value	Significant	p_{corr} value	Significant
BioSNAP	XGBoost	5.63×10^{-9}	True	4.06×10^{-9}	True
	MolTrans[22]	1.70×10^{-7}	True	4.48×10^{-6}	True
	Kang *et al.*[4]	6.49×10^{-5}	True	3.37×10^{-5}	True
	DLM-DTI[26]	4.26×10^{-6}	True	3.65×10^{-5}	True
	ConPLex[36]	—	—	5.77×10^{-10}	True
BindingDB	XGBoost	3.45×10^{-7}	True	1.89×10^{-6}	True
	MolTrans[22]	3.45×10^{-7}	True	1.89×10^{-6}	True
	Kang *et al.*[4]	1.70×10^{-6}	True	1.19×10^{-5}	True
	DLM-DTI[26]	1.58×10^{-4}	True	1.89×10^{-6}	True
	ConPLex[36]	—	—	2.84×10^{-5}	True
DAVIS	XGBoost	6.78×10^{-7}	True	5.79×10^{-8}	True
	MolTrans[22]	2.89×10^{-7}	True	3.74×10^{-5}	True
	Kang *et al.*[4]	6.78×10^{-7}	True	1.41×10^{-6}	True
	DLM-DTI[26]	6.78×10^{-7}	True	3.00×10^{-5}	True
	ConPLex [36]	—	—	1.82×10^{-4}	True

Tab. S6: Statistical testing of ablation benchmark with BarlowDTI against other models using Kang *et al.* splits.[4] Five replicates each were performed. Two-sided Welch's t-test,[33, 34] $\alpha = 0.0001$ with Benjamini-Hochberg[35] multiple test correction was applied. (o.: optimised; n.o.: non-optimised)

**Comparison**		**PR AUC**	
Model 1	Model 2	p_{corr} value	Significant
XGBoost	XGBoost o.	1.47×10^{-7}	True
XGBoost	BarlowDTI n.o.	2.37×10^{-23}	True
XGBoost	BarlowDTI	6.32×10^{-25}	True
XGBoost o.	BarlowDTI n.o.	1.64×10^{-10}	True
XGBoost o.	BarlowDTI	9.48×10^{-15}	True
BarlowDTI n.o.	BarlowDTI	1.70×10^{-15}	True

Tab. S7: Statistical testing of benchmarking BarlowDTI against other models using Kang *et al.* splits.[4] For XGBoost five replicates were performed. Two-sided Welch's t-test,[33, 34] $\alpha = 0.05$ with Benjamini-Hochberg[35] multiple test correction was applied.

**Comparison**		**PR AUC**	
Model 1	Model 2	p_{corr} value	Significant
XGBoost	DLM-DTI	0.1452	False
XGBoost	MolTrans	0.1452	False
XGBoost	Kang *et al.*	0.1452	False
DLM-DTI	MolTrans	0.7970	False
DLM-DTI	Kang *et al.*	0.7970	False
MolTrans	Kang *et al.*	0.7970	False

Tab. S8: Statistical testing of benchmarking BarlowDTI against other models in PDBBind v2016 and v2020. Five replicates were performed. Two-sided Welch's t-test,[33, 34] $\alpha = 0.05$ with Benjamini-Hochberg[35] multiple test correction was applied.

**Comparison**	p_{corr} value for PDBBind v2016		
BarlowDTI vs.	MAE	ρ	CI
XGBoost	6.33×10^{-4}	1.50×10^{-3}	4.17×10^{-3}
SMINA	1.67×10^{-10}	1.95×10^{-8}	3.94×10^{-9}
GNINA	6.33×10^{-4}	5.40×10^{-4}	3.58×10^{-3}
dMaSIF	1.48×10^{-1}	1.12×10^{-3}	2.70×10^{-3}
GraphDTA	6.29×10^{-2}	6.82×10^{-4}	3.06×10^{-4}
TransCPI	1.29×10^{-2}	1.50×10^{-3}	1.67×10^{-4}
MolTrans	6.33×10^{-4}	6.01×10^{-4}	3.03×10^{-4}
DrugBAN	2.43×10^{-6}	5.83×10^{-1}	2.99×10^{-2}
WGNN-DTA	1.48×10^{-5}	2.19×10^{-5}	2.37×10^{-5}
STAMP-DPI	3.19×10^{-1}	1.50×10^{-3}	6.26×10^{-6}
PSICHIC	6.33×10^{-4}	2.48×10^{-3}	2.70×10^{-3}
	p_{corr} value for PDBBind v2020		
BarlowDTI vs.	MAE	ρ	CI
XGBoost	2.19×10^{-7}	1.07×10^{-5}	3.61×10^{-6}
SMINA	8.01×10^{-9}	6.05×10^{-6}	1.20×10^{-7}
GNINA	2.81×10^{-12}	3.58×10^{-3}	3.61×10^{-6}
dMaSIF	2.33×10^{-8}	1.44×10^{-6}	6.77×10^{-8}
GraphDTA	5.31×10^{-6}	1.44×10^{-6}	6.77×10^{-8}
TransCPI	7.85×10^{-8}	1.07×10^{-5}	9.87×10^{-8}
MolTrans	7.62×10^{-7}	2.73×10^{-2}	1.54×10^{-7}
DrugBAN	3.89×10^{-7}	9.57×10^{-7}	6.77×10^{-8}
WGNN-DTA	1.96×10^{-6}	1.25×10^{-5}	9.87×10^{-8}
STAMP-DPI	7.05×10^{-6}	8.65×10^{-6}	6.77×10^{-8}
PSICHIC	5.85×10^{-8}	1.72×10^{-6}	7.41×10^{-8}

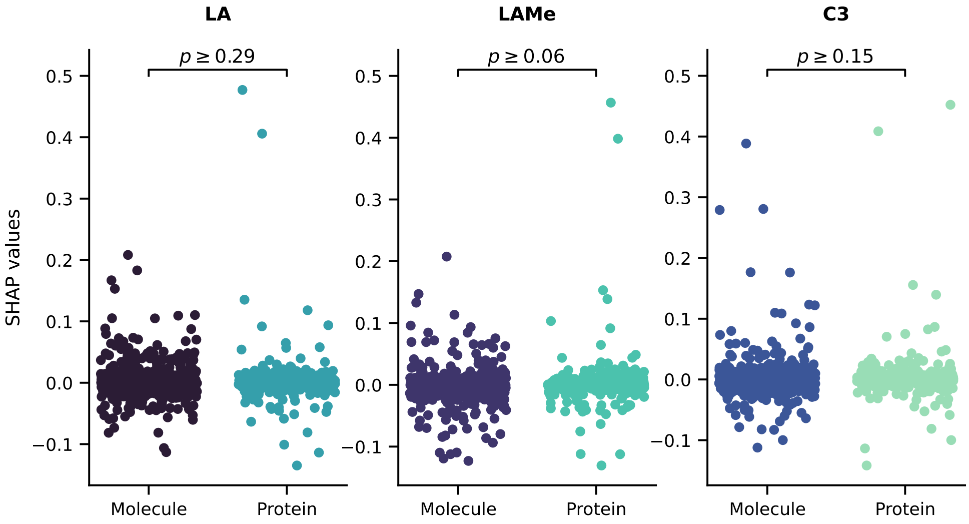

Fig. S5: SHAP values of BarlowDTI$_{XXL}$ input modalities. No significant change in distribution could be shown, independent of the ligand molecule, case study based on the Dienemann *et al.* publication.[37] A two-sided Wilcoxon[38] signed-rank test was applied and respective *p*-values are presented within the figure.

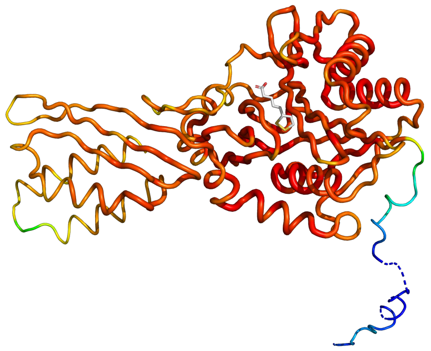

Fig. S6: B-factor visualisation of RF-AA[32] prediction of LIPT1.

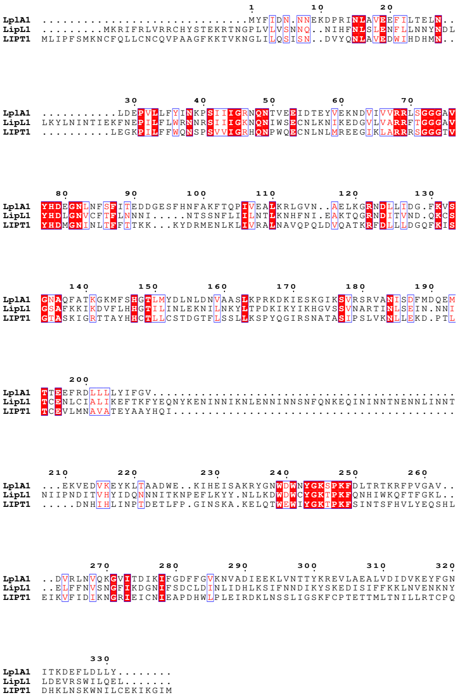

Fig. S7: Sequence alignment of lplA1, LipL1 and LIPT1.

References

[1] Zitnik, M., Sosič, R., Maheshwari, S. & Leskovec, J. BioSNAP Datasets: Stanford biomedical network dataset collection. http://snap.stanford.edu/biodata (2018).

[2] Liu, T., Lin, Y., Wen, X., Jorissen, R. N. & Gilson, M. K. BindingDB: A web-accessible database of experimentally determined protein–ligand binding affinities. *Nucleic Acids Research* **35**, D198–D201 (2007).

[3] Davis, M. I. *et al.* Comprehensive analysis of kinase inhibitor selectivity. *Nature Biotechnology* **29**, 1046–1051 (2011).

[4] Kang, H. *et al.* Fine-tuning of BERT Model to Accurately Predict Drug–Target Interactions. *Pharmaceutics* **14**, 1710 (2022).

[5] Li, S. *et al.* Structure-aware Interactive Graph Neural Networks for the Prediction of Protein-Ligand Binding Affinity (2021).

[6] Su, M. *et al.* Comparative Assessment of Scoring Functions: The CASF-2016 Update. *Journal of Chemical Information and Modeling* **59**, 895–913 (2019).

[7] Lu, W. *et al.* TANKBind: Trigonometry-Aware Neural NetworKs for Drug-Protein Binding Structure Prediction (2022).

[8] Stärk, H., Ganea, O., Pattanaik, L., Barzilay, D. R. & Jaakkola, T. EquiBind: Geometric Deep Learning for Drug Binding Structure Prediction (2022).

[9] Koh, H. Y., Nguyen, A. T. N., Pan, S., May, L. T. & Webb, G. I. Physicochemical graph neural network for learning protein–ligand interaction fingerprints from sequence data. *Nature Machine Intelligence* **6**, 673–687 (2024).

[10] Carhart, R. E., Smith, D. H. & Venkataraghavan, R. Atom pairs as molecular features in structure-activity studies: Definition and applications. *Journal of Chemical Information and Computer Sciences* **25**, 64–73 (1985).

[11] Rogers, D. & Hahn, M. Extended-Connectivity Fingerprints. *Journal of Chemical Information and Modeling* **50**, 742–754 (2010).

[12] Hall, L. H. & Kier, L. B. Electrotopological State Indices for Atom Types: A Novel Combination of Electronic, Topological, and Valence State Information. *Journal of Chemical Information and Computer Sciences* **35**, 1039–1045 (1995).

[13] Durant, J. L., Leland, B. A., Henry, D. R. & Nourse, J. G. Reoptimization of MDL Keys for Use in Drug Discovery. *Journal of Chemical Information and Computer Sciences* **42**, 1273–1280 (2002).

[14] Capecchi, A., Probst, D. & Reymond, J.-L. One molecular fingerprint to rule them all: Drugs, biomolecules, and the metabolome. *Journal of Cheminformatics*

12, 43 (2020).

[15] Landrum, G. *et al.* Rdkit/rdkit: 2020_03_1 (Q1 2020) Release. Zenodo (2020).

[16] Elnaggar, A. *et al.* ProtTrans: Toward Understanding the Language of Life Through Self-Supervised Learning. *IEEE Transactions on Pattern Analysis and Machine Intelligence* **44**, 7112–7127 (2022).

[17] Heinzinger, M. *et al.* Bilingual Language Model for Protein Sequence and Structure (2024).

[18] Koes, D. R., Baumgartner, M. P. & Camacho, C. J. Lessons Learned in Empirical Scoring with smina from the CSAR 2011 Benchmarking Exercise. *Journal of Chemical Information and Modeling* **53**, 1893–1904 (2013).

[19] McNutt, A. T. *et al.* GNINA 1.0: Molecular docking with deep learning. *Journal of Cheminformatics* **13**, 1–20 (2021).

[20] Sverrisson, F., Feydy, J., Correia, B. E. & Bronstein, M. M. Fast end-to-end learning on protein surfaces (2020).

[21] Nguyen, T. *et al.* GraphDTA: Predicting drug–target binding affinity with graph neural networks. *Bioinformatics* **37**, 1140–1147 (2021).

[22] Huang, K., Xiao, C., Glass, L. M. & Sun, J. MolTrans: Molecular Interaction Transformer for drug–target interaction prediction. *Bioinformatics* **37**, 830–836 (2021).

[23] Bai, P., Miljković, F., John, B. & Lu, H. Interpretable bilinear attention network with domain adaptation improves drug–target prediction. *Nature Machine Intelligence* **5**, 126–136 (2023).

[24] Jiang, M. *et al.* Sequence-based drug-target affinity prediction using weighted graph neural networks. *BMC Genomics* **23**, 449 (2022).

[25] Wang, P. *et al.* Structure-Aware Multimodal Deep Learning for Drug–Protein Interaction Prediction. *Journal of Chemical Information and Modeling* **62**, 1308–1317 (2022).

[26] Lee, J., Jun, D. W., Song, I. & Kim, Y. DLM-DTI: A dual language model for the prediction of drug-target interaction with hint-based learning. *Journal of Cheminformatics* **16**, 1–12 (2024).

[27] Kim, S. *et al.* PubChem 2023 update. *Nucleic Acids Research* **51**, D1373–D1380 (2023).

[28] de Cárcer, G. *et al.* Plk1 overexpression induces chromosomal instability and suppresses tumor development. *Nature Communications* **9**, 3012 (2018).

[29] Lee, K. S., Burke, T. R., Park, J.-E., Bang, J. K. & Lee, E. Recent Advances and New Strategies in Targeting Plk1 for Anticancer Therapy. *Trends in Pharmacological Sciences* **36**, 858–877 (2015).

[30] Jain, A. N., Cleves, A. E. & Walters, W. P. Deep-Learning Based Docking Methods: Fair Comparisons to Conventional Docking Workflows (2024). 2412.02889.

[31] Abramson, J. *et al.* Accurate structure prediction of biomolecular interactions with AlphaFold 3. *Nature* **630**, 493–500 (2024).

[32] Krishna, R. *et al.* Generalized biomolecular modeling and design with RoseTTAFold All-Atom. *Science* **384**, eadl2528 (2024).

[33] WELCH, B. L. THE GENERALIZATION OF 'STUDENT'S' PROBLEM WHEN SEVERAL DIFFERENT POPULATION VARLANCES ARE INVOLVED. *Biometrika* **34**, 28–35 (1947).

[34] Virtanen, P. *et al.* SciPy 1.0: Fundamental algorithms for scientific computing in Python. *Nature Methods* **17**, 261–272 (2020).

[35] Benjamini, Y. & Hochberg, Y. Controlling the False Discovery Rate: A Practical and Powerful Approach to Multiple Testing. *Journal of the Royal Statistical Society. Series B (Methodological)* **57**, 289–300 (1995).

[36] Singh, R., Sledzieski, S., Bryson, B., Cowen, L. & Berger, B. Contrastive learning in protein language space predicts interactions between drugs and protein targets. *Proceedings of the National Academy of Sciences* **120**, e2220778120 (2023).

[37] Dienemann, J.-N. *et al.* A Chemical Proteomic Strategy Reveals Inhibitors of Lipoate Salvage in Bacteria and Parasites. *Angewandte Chemie International Edition* **62**, e202304533 (2023).

[38] Wilcoxon, F. Individual Comparisons by Ranking Methods. *Biometrics Bulletin* **1**, 80–83 (1945).
